# Vaccination of Mice Using the West Nile Virus E-Protein in a DNA Prime-Protein Boost Strategy Stimulates Cell-Mediated Immunity and Protects Mice against a Lethal Challenge

**DOI:** 10.1371/journal.pone.0087837

**Published:** 2014-02-04

**Authors:** Marina De Filette, Silke Soehle, Sebastian Ulbert, Justin Richner, Michael S. Diamond, Alessandro Sinigaglia, Luisa Barzon, Stefan Roels, Julianna Lisziewicz, Orsolya Lorincz, Niek N. Sanders

**Affiliations:** 1 Laboratory of Gene Therapy, Faculty of Veterinary Sciences, Ghent University, Merelbeke, Belgium; 2 Institute of Virology, University of Zurich, Zurich, Switzerland; 3 Department of Immunology, Fraunhofer Institute for Cell Therapy and Immunology, Leipzig, Germany; 4 Departments of Medicine, Molecular Microbiology and Pathology and Immunology, Washington University School of Medicine, St. Louis, Missouri, United States of America; 5 Department of Molecular Medicine, University of Padova, Padova, Italy; 6 Operational Direction Interactions and Surveillance, Veterinary and Agrochemical Research Centre (CODA/CERVA), Brussels, Belgium; 7 Genetic Immunity, Budapest, Hungary and McLean, Virginia, United States of America; Fondazione IRCCS Policlinico San Matteo, Italy

## Abstract

West Nile virus (WNV) is a mosquito-borne flavivirus that is endemic in Africa, the Middle East, Europe and the United States. There is currently no antiviral treatment or human vaccine available to treat or prevent WNV infection. DNA plasmid-based vaccines represent a new approach for controlling infectious diseases. In rodents, DNA vaccines have been shown to induce B cell and cytotoxic T cell responses and protect against a wide range of infections. In this study, we formulated a plasmid DNA vector expressing the ectodomain of the E-protein of WNV into nanoparticles by using linear polyethyleneimine (lPEI) covalently bound to mannose and examined the potential of this vaccine to protect against lethal WNV infection in mice. Mice were immunized twice (prime – boost regime) with the WNV DNA vaccine formulated with lPEI-mannose using different administration routes (intramuscular, intradermal and topical). In parallel a heterologous boost with purified recombinant WNV envelope (E) protein was evaluated. While no significant E-protein specific humoral response was generated after DNA immunization, protein boosting of DNA-primed mice resulted in a marked increase in total neutralizing antibody titer. In addition, E-specific IL-4 T-cell immune responses were detected by ELISPOT after protein boost and CD8^+^ specific IFN-γ expression was observed by flow cytometry. Challenge experiments using the heterologous immunization regime revealed protective immunity to homologous and virulent WNV infection.

## Introduction

West Nile virus (WNV) is a single-stranded positive polarity enveloped RNA virus and member of the Flavivirus genus of the *Flaviviridae* family. WNV is transmitted in a natural cycle between birds and mosquitoes [1] and causes morbidity and mortality in birds, horses, humans and some other vertebrate animals. In humans, WNV infections usually remains asymptomatic or causes a mild undifferentiated febrile illness called West Nile fever [2]. However, in some individuals, mainly in the immunocompromised or elderly [3], WNV infection can develop into severe, potentially life-threatening neuroinvasive disease. WNV has circulated in the United States since 1999 [4] and subsequently spread across continental North America, the Caribbean and South America [5]. It was soon recognized as one of the most widely distributed flaviviruses, with its geographic range including Africa [6], the Middle East [6] western Asia [6], Europe [6] and Australia [7]. Several vaccines, including conventional killed [8], DNA plasmid [9] and recombinant vectored vaccines [10], [11], are commercially available to prevent WNV infection of horses and exotic birds. So far, no vaccine has been approved for human use and mosquito control is the only available strategy to combat the spread of this disease in humans. Since there is also no treatment for WNV infection available, there is an urgent need for effective vaccines to prevent WNV infection in humans.

DNA vaccines were introduced more than 20 years ago [12] and have been applied to a range of infectious and malignant diseases. Developments in this field have advanced greatly over the years, and DNA vaccines against various pathogens (influenza [13], [14], HPV [15], [16], HIV [17]) have entered human phase I and II clinical trials [18]. Importantly, like live vaccines, DNA vaccines induce a combined humoral and cellular immunity against pathogens. In addition, DNA vaccines can circumvent many of the problems associated with recombinant protein-based vaccines, such as high cost of production, difficulties in purification, incorrect folding of antigen and poor induction of CD8^+^ cells. However the efficacy of genetic vaccines *in vivo* has not always been satisfactory. Many approaches have been used in an attempt to improve the efficacy of DNA vaccines such as codon and promoter optimization [19]–[21], addition of adjuvants [22], [23], formulation with cationic liposomes [24] or polymers [25] and the use of heterologous prime-boost regimes [26], [27]. Previously, the group of Schneeweiss *et al.* [28] designed a pDNA vaccine that expressed only the E-protein ectodomain. In a preliminary study they showed that the pDNA vaccine was protective against WNV strain IS-98-ST1 after a single intramuscular electrogene transfer in mice. They also demonstrated that it was possible to boost the immunogenicity of the electroporated DNA vaccine with recombinant domain III of the E-protein. This domain is important for induction of neutralizing antibodies in mice but the human antibody response is skewed away from DIII towards less-neutralizing epitopes in domains I and II and therefore E-protein, which is used in this work, is more appropriate for boosting. In the preliminary experiment of Schneeweiss et al., the immune response was only briefly monitored and electroporation was used to deliver the pDNA vaccine [28]. Since electroporation requires specialized equipment this administration method is not practical for vaccination of humans on a large scale. Therefore, we explored the use of mannose modified linear polyethyleneimine (lPEIm also called DermaVir) as carrier for this WNV DNA vaccine. DermaVir has been designed for topical application of DNA based vaccines [29]. Currently, there is no information on the immune response of DermaVir based DNA vaccines after other administration routes. In this study DermaVir was used to formulate the WNV pDNA vaccine and the resulting WNV-DermaVir nanoparticles were administered topically, intradermally or intramuscularly.

A DNA prime/DNA boost and also a DNA prime/protein boost vaccination schedule were evaluated. For successful DNA vaccination, the selected delivery system must deliver the DNA efficiently into the target tissue and cells with the least toxicity and without inducing a harmful immune response. Cationic polymers are promising gene-delivery vehicles [30], [31] and among them polyethylenimine (PEI) is one of the most widely used [32]. Polyethylenimine (PEI) is a cationic polymer containing repeating units of ethyleneimine (-CH_2_CH_2_NH-). Linear polyethyleneimines (lPEI) contain secondary and primary (at the ends) amines, in contrast to branched PEIs which contain primary, secondary and tertiary amino groups. lPEI electrostatically condenses nucleic acids and forms stable nanoparticles so-called polyplexes [33]. Its free amine groups can be used to conjugate cell binding ligands such as mannose [34]–[36], which binds to the mannose receptor present on the surface of antigen presenting cells (APCs) e.g. dendritic cells (DCs) and macrophages and liver endothelial cells [37]. In addition, mannose binding lectin, which belongs to the family of calcium-dependent collagenous lectins (collectins), is an important protein of the innate immune system that activates the complement system [38]. Therefore, as our WNV-DermaVir nanoparticles contain mannose they may activate the lectin pathway of the complement and undergo complement-mediated phagocytosis. However, Lorincz *et al.* investigated the activation of the complement and they could not detect nor exclude complement activation by DermaVir [39]. In this study, we demonstrate that DNA vaccination using lPEI-mannose (LPEIm) as delivery vehicle failed to induce a measurable humoral immune response by itself, but upon protein boosting we noticed a marked increase in overall and neutralizing antibody titers against WNV. Importantly, boosted mice were fully protected against a lethal challenge with WNV.

## Materials and Methods

### WNV DNA Vaccine, Control Plasmid and E-protein

The construction of the WNV DNA vaccine, pT-WNV-E, has been described previously [28]. To generate a control plasmid, the sequence coding for the E-ectodomain in pT-WNV-E was replaced by the coding sequence for EGFP. The WNV E ectodomain (amino acid residues 1 to 404) of the New York 1999 strain was amplified from an infectious cDNA clone, and cloned into the pET21a bacterial expression plasmid. WNV E protein was expressed in Escherichia coli and purified by using an oxidative refolding protocol, as described in detail previously [40]. Recombinant WNV domain DIII was produced as described in [30].

### Preparation and Characterization of the WNV-DermaVir Nanoparticles

Linear polyethyleneimine-mannose (lPEIm) was prepared as previously reported by Lorincz [39] covalently coupling of 3% mannose (calculated on the nitrogen content of the polymer) to 22 kDa lPEI (manufactured by Genetic Immunity). DNA/PEIm nanoparticles containing the WNV DNA vaccine were prepared at a N/P ratio of 4 as described earlier [39]. Briefly, one volume of WNV DNA vaccine was mixed with 3 volumes of TEAM buffer (10% mannitol containing 10 mM triethanolamine buffer pH 7.6), and 1 volume lPEIm was mixed with 3 volumes of TEAM buffer. Subsequently, the diluted PEIm was mixed with an equal volume of the diluted WNV DNA vaccine. Nanoparticle formation was allowed to proceed for 30 minutes after mixing. The final WNV DNA vaccine concentration in the lPEIm-DNA nanoparticles (WNV-DermaVir) was 0.125 µg/µl. After the preparation of the nanoparticles the following quality control assays were performed. The nanoparticles as well as the individual components were measured by UV-Vis spectrophotometry (Jasco V-630 UV-vis spectrophotometer) against purified water. Hyperchromicity of the nanoparticle was calculated following the equation : Hyperchromicity % = 100×(A260_DermaVir_ – ∑A260_components_*)/(∑A260_components_) with *∑A260_components_ = A260_pDNA_+A260_PEIm_+A260_TEAM._ Additionally, the particle size was measured via dynamic light scattering after diluting the samples with purified water using the ZetaPALS instrument (Brookhaven). Zeta potential was measured via electrophoretic light scattering using the same instrument as for the particle size measurements.

### Mice

Specified pathogen-free female BALB/c mice were obtained from Janvier Labs (Le genest-Saint-Isle, France) and immunized at the age of eight weeks. The animals were housed in a temperature-controlled environment with 12 h light/dark cycles, and received food and water ad libitum. All animal experiments were authorized by the Institutional Ethics Committee on Experimental Animals and performed under conditions specified by law (European Directive and Belgian Royal Decree of November 14, 1993). The protocol was approved by the ethics committee of the faculty of Veterinary Medicine of Ghent University (Permit Number: EC2012/124). All manipulations were performed under isoflurane anesthesia, and all efforts were made to minimize suffering.

### Vaccination Procedures

**Figure 1:**
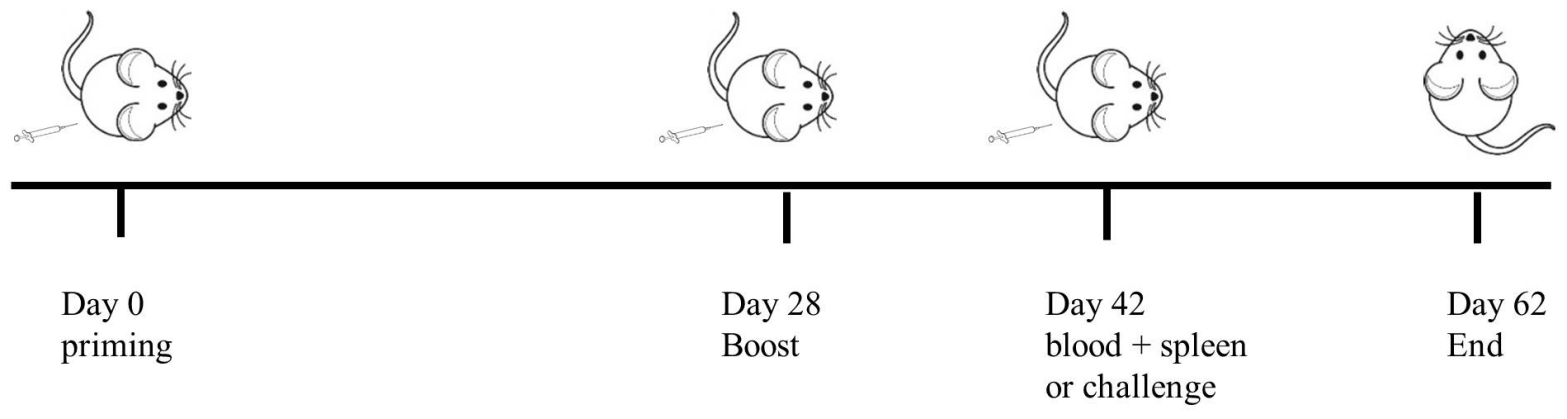
A graphical scheme with a timeline of the mice experiments.

A graphical scheme with a timeline of the mice experiments is shown in Figure 1. One microgram of plasmid pTWNV-E or eGFP control plasmid was formulated with lPEIm. The resulting nanoparticles, called WNV-DermaVir nanoparticles, were subsequently administered intradermally (ID), intramuscularly (IM) or topically to the mice. For topical application, the hair of the dorsum was removed by shaving and the skin was exfoliated with a sponge as previously described [29]. Finally, tape stripping was performed and 80 µl WNV-DermaVir nanoparticles (containg 1 µg pTWNV-E) was applied on the entire tape stripped area. After air-drying, mice were returned to the corresponding cages. Mice were boosted four weeks later either via an IM injection of the same dose of WNV-DermaVir nanoparticles or via subcutanous (s.c.) injection of recombinant E-protein ([28]) formulated with Matrix-M1 (a kind gift from Linda Stertman (Isconova AB)).

### Determination of Serum Antibody Levels and Virus Neutralization Antibody Titers

Two weeks after the boost, blood samples were collected by cardiac puncture. Blood was allowed to clot for 60 min at 37°C, and serum was obtained by combining the supernatant from two successive centrifugations. The titers of E- and DIII-specific IgG1 and IgG2a antibodies in the serum were determined by ELISA in 96-well Maxisorp immuno-plates (Nunc) coated overnight with recombinant E-protein or DIII (1 µg/ml in carbonate buffer, 100 µl/well, 4°C) ([28]). After coating, the plates were washed three times with phosphate buffered saline (PBS) containing 0.1% Tween-20 and blocked with 3% skim milk in PBS. Next, three-fold serial dilutions of mouse serum, starting with a 1/100 dilution, were incubated for 1 h while shaking at room temperature. After washing goat-derived anti-mouse serum conjugated with horseradish peroxidase specific for mouse isotypes IgG1 or IgG2a (Southern Biotechnology Associates) and tetramethylbenzidine substrate (BD Biosciences) were used to determine specific antibody titers. Antibody titers are defined as the reciprocal of the highest dilution with an OD_450_ that is at least double the OD_450_ of preimmune serum samples. The titer of neutralizing antibody in serum against WNV was quantitated by a focus reduction neutralization assay in Vero cells as described previously [41].

### Detection of Mouse IL-4 T Cell Responses by ELISPOT

This analysis was performed with splenocytes isolated 14 days after boosting. The spleens of four to five mice per group were isolated aseptically and single-cell suspensions were prepared. Splenocyte suspensions were depleted of red blood cells using ammonium chloride hypotonic lysis and passed through a 70-µm cell strainer. For ELISPOT analysis, sterile 96-well Maxisorp immuno-plates were coated with anti-IL-4 monoclonal antibodies (Biolegend) and blocked with sterile PBS containing 1% BSA for 1 h at 37°C. Next, 3×10^5^ splenocytes were plated in 100 µl of culture medium and stimulated during 16 h with culture medium only (negative control), 5 µg/mL Phytohemagglutinin (PHA, positive control IL-4, Sigma–Aldrich), 4 µg/mL Concanavalin A (ConA, positive control IFN-γ, Sigma–Aldrich) or 2 µg/ml of purified E-protein. After stimulation the plates were washed two times with PBS and four times with PBS containing 0,05% Tween-20. IL-4 trapped on the plates was detected by a biotinylated monoclonal anti-IL-4 antibody (Biolegend). Subsequent incubation with GABA-conjugated streptavidin (U-Cytech Biosciences) was used to develop silver spots at places were immune cells secreted IL-4 during stimulation with E-protein or ConA. Splenocytes from each mouse were analyzed in triplicate and the spots were counted using the Bioreader 5000 (BIO-SYS).

### Intracellular Cytokine Staining (ICS)

Splenocytes were prepared as described above. Subsequently, the splenocytes of four to five mice per treatment group were pooled and incubated for 6 h at 37°C with 2 µg E-protein, medium or conA in the presence of 5 µg/ml brefeldin A (Biolegend). After incubation the cells were washed and blocked with Fc block (anti-mouse FcγRI/III; BD Pharmingen, BD Biosciences) and incubated 30 min in the presence of a saturating dose of surface antibodies against CD4 or CD8. After washing, the cells were fixed and permeabilized, and an antibody against IFN-γ was added for 30 min. After washing, the cells were resuspended in staining solution (Biolegend) and analyzed using the Accuri C6 (BD Biosciences). Flow cytometry analysis was performed by collecting 5×10^4^ events and gates were set on lymphocyte population based on forward and side scatter, followed by marker positioning to denote fluorescence greater than that of control stained or unstained cells.

### Histopathology and Toxicology

Fourteen days after the priming, mice that received WNV-DermaVir nanoparticles topically were euthanized and skin samples were collected, fixed in 4% paraformaldehyde, embedded in paraffin wax, sectioned at 5 µm, and stained with hematoxylin-eosin. Slides were examined in a blinded fashion using an Olympus BX 50 microscope (Olympus) and micrographs were made.

Serum alanine transaminase (ALT) and total serum bilirubin levels were determined by using commercial kits produced by Bioo Scientific following the manufacturer’s instruction. Serum samples were analyzed two weeks after priming.

### WNV Challenge in Mice

To evaluate the protective efficacy of the vaccine, mice were challenged with a lineage 1 West Nile virus (Italy/2009) two weeks after boosting. The Italy/2009 strain was isolated in Vero cells in 2009 from an asymptomatic blood donor screened by nucleic acid testing for WNV infection, who was resident in Rovigo, Italy. The full genome of the Italy/2009 isolate (GenBank accession number GU011992.2) had 99% nucleotide sequence identity with other WNV isolates collected during the outbreaks that occurred in the Po river area in northern Italy in 2008 and 2009 [42]. The Italy/2009 stock used in the experiments reported in this study was passage 2, cultured in Vero E6 cells. Pathogenicity of the Italy/2009 isolate was compared with that of other European WNV lineage 1 and lineage 2 strains in a mouse model, which demonstrated that the Italy/2009 strain is highly neurovirulent and lethal [43]. The challenge dose (10^4^ TCID_50_) was administered intraperitoneally in a volume of 200 µl to the mice. Mice were monitored at least daily for 20 days with a scoring system based on loss of body weight, physical condition and behavior. They were monitored daily and twice daily if abnormalities were observed in the morning. Animals were euthanized once they reached a certain scoring number.

### Determination of Viral RNA Levels in Blood and Brain

Blood was collected two days after WNV challenge. Mice from each group were euthanized at their endpoint or 20 days after challenge. The brains were removed and homogenized in lysis buffer and proteinase K at 56°C for 2 h. The extracts were transferred to centrifuge tubes and cell debris was pelleted for 10 min at 400 g and 4°C. The levels of viral RNA were determined by real-time RT-PCR using the oligonucleotide primers and TaqMan probe targeting the WNV E-gene designed by Lanciotti *et al.* (32). For detection of WNV RNA in blood, nucleic acids were purified from plasma samples by using a MagNA Pure 96 System (Roche) and eluted in a final volume of 100 µl. Then, 10 microliters of RNA was combined with Superscript(r) One Step RT-PCR System reagents (Agpath-ID™, Life Technologies), primers and probe in a 20-µl total reaction volume and amplified in a 7900HT Real-Time PCR System (Life Technologies,).

### Statistical Analysis

Survival curves were plotted and evaluated statistically according to Kaplan–Meier by using the GraphPad Prism 5 software. Morbidity parameters and antibody titers between more than two experimental groups were compared with Tukey’s test for multiple comparison of means. The statistical package SPSS was used.

## Results

### Characterization of WNV-DermaVir Nanoparticles

Linear PEI (lPEI) possesses DNA binding and condensing activity together with a high pH buffering capacity that is believed to protect DNA from degradation and to enhance escape from the endosomal compartment. In addition, by employing mannose-lPEI conjugates we can direct the delivery of DNA vaccine towards dendritic cells. The size and the zeta potential of the WNV-DermaVir nanoparticles containing the WNV DNA vaccine ranged around 114 nm and 8 mV, respectively (Table 1). The percentage hyperchromicity, which is a measure of the degree of pDNA compaction, ranged around 3.2% [39].

**Table 1:** Particle size and zeta potential of three batches of DermaVir nanoparticles containing the WNV DNA vaccine.

	Average size (nm) ± SD (nm)	Average zeta potential (mV) ± SD (mV)
**NP1**	112±3	10.6±2.6
**NP2**	113±7	9.4±5.1
**NP3**	117±3	4.4±2.2
**Total average**	**114±5**	**8.2±4.2**

Abbreviations: SD, standard deviation; NP,nanoparticle. Three samples were measured per batch.

### Immunization with WNV-DermaVir Nanoparticles Followed by a Protein Boost Generate Titers of Th2 E-protein Specific Antibodies

One of the parameters that play a role in the immune response elicited by nanoparticle based delivery systems for DNA vaccines is the route of administration. To study the effect of the administration route on the immunogenicity, mice were vaccinated with the WNV-DermaVir nanoparticles via three different routes: intramuscular, intradermal and topical (Table 2).

**Table 2:** Vaccination regime and BALB/c mice groups.

Prime-boost	DNA prime (day 1)	DNA boost (day 28)	protein boost (day 28)
**IM-IM**	5 mice IM 1 µg	5 mice IM 1 µg	/
**IM-E**	5 mice IM 1 µg	/	5 mice 10 µg E+Matrix-M1
**Top-IM**	5 mice top 1 µg	5 mice IM 1 µg	/
**Top-E**	5 mice top 1 µg	/	5 mice 10 µg E+Matrix-M1
**ID-ID**	4 mice ID 1 µg	4 mice ID 1 µg	
**ID-E**	4 mice ID 1 µg	/	4 mice 10 µg E+Matrix-M1
**−/E**	No prime	/	5 mice 10 µg E+Matrix-M1

Abbreviations; IM: intramuscular, Top: topical, ID: intradermal, E: WNV E-protein (ectodomain).

At 28 days, serum was collected. Subsequently several of the animals were boosted homologously or heterologously with WNV-DermaVir nanoparticles or E-protein respectively. Two weeks after the boost, serum samples were collected for determining levels of anti-E antibody by ELISA.

**Figure 2:**
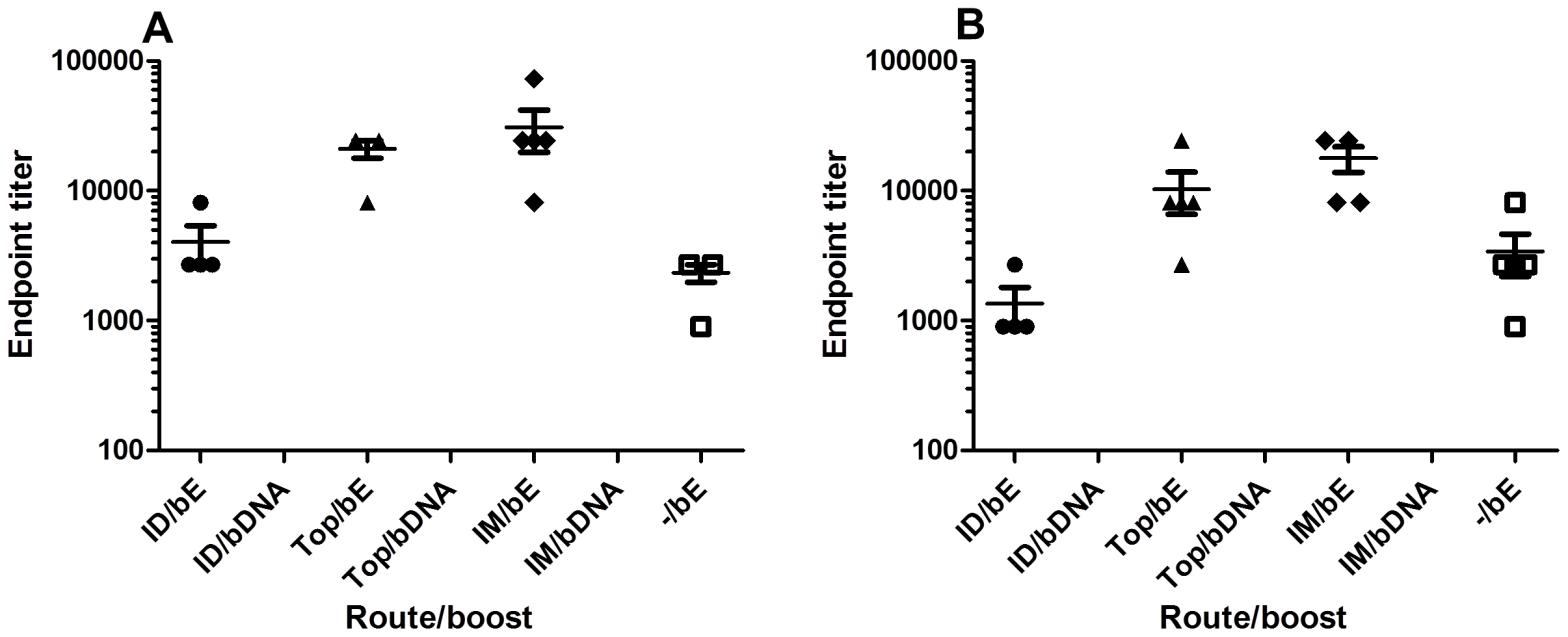
Detection of serum antibodies to the E-protein. IgG1 (A) and IgG2a (B) titers of mice that were ID (4 mice), IM (5 mice), Topically (5 mice) or not primed (−/bE, 5 mice) with WNV-DermaVir nanoparticles and boosted either with WNV-DermaVir nanoparticles (IM) or E-protein combined with Matrix-M1 adjuvant (s.c.). IgG1 (A) and IgG2a (B) titers were determined by ELISA. Abbreviations: bE: boost E-protein, bDNA: boost WNV-DermaVir nanoparticles.

The anti-WNV levels of virus-specific IgG1 and IgG2a antibodies in serum were measured by ELISA. Notably, at 28 days, we failed to detect anti-E antibodies in mice primed with WNV-DermaVir nanoparticles. After boosting these mice with WNV-DermaVir nanoparticles, we still did not detect anti-E antibodies in their sera (Figure 2). In contrast, mice primed with WNV-DermaVir nanoparticles and boosted with E-protein developed measurable IgG1 (Figure 2A) and IgG2a (Figure 2B) titers against the WNV-E protein. Mice primed with WNV-DermaVir nanoparticles and boosted with the E-protein also showed a T-helper (Th)-2 skewed response. Indeed, the IgG1/IgG2a ratio’s for ID, IM and Top administration were respectively 3, 1.7 and 2.1. The highest antibody titers were obtained in the groups primed IM or topically with the nanoparticles. The mean difference between the ID vaccinated group and the IM vaccinated group was significant at the 0.05 level (Tukey’s test). When a single vaccination with E-protein (without priming) was given, the IgG1 (Figure 2A) and IgG2a (Figure 2B) titers were lower and a Th1 shifted response was induced. The titers of the unprimed mice were statistical significantly lower compared to the titers obtained for the IM primed mice (P<0.05, Tukey’s test).

**Figure 3:**
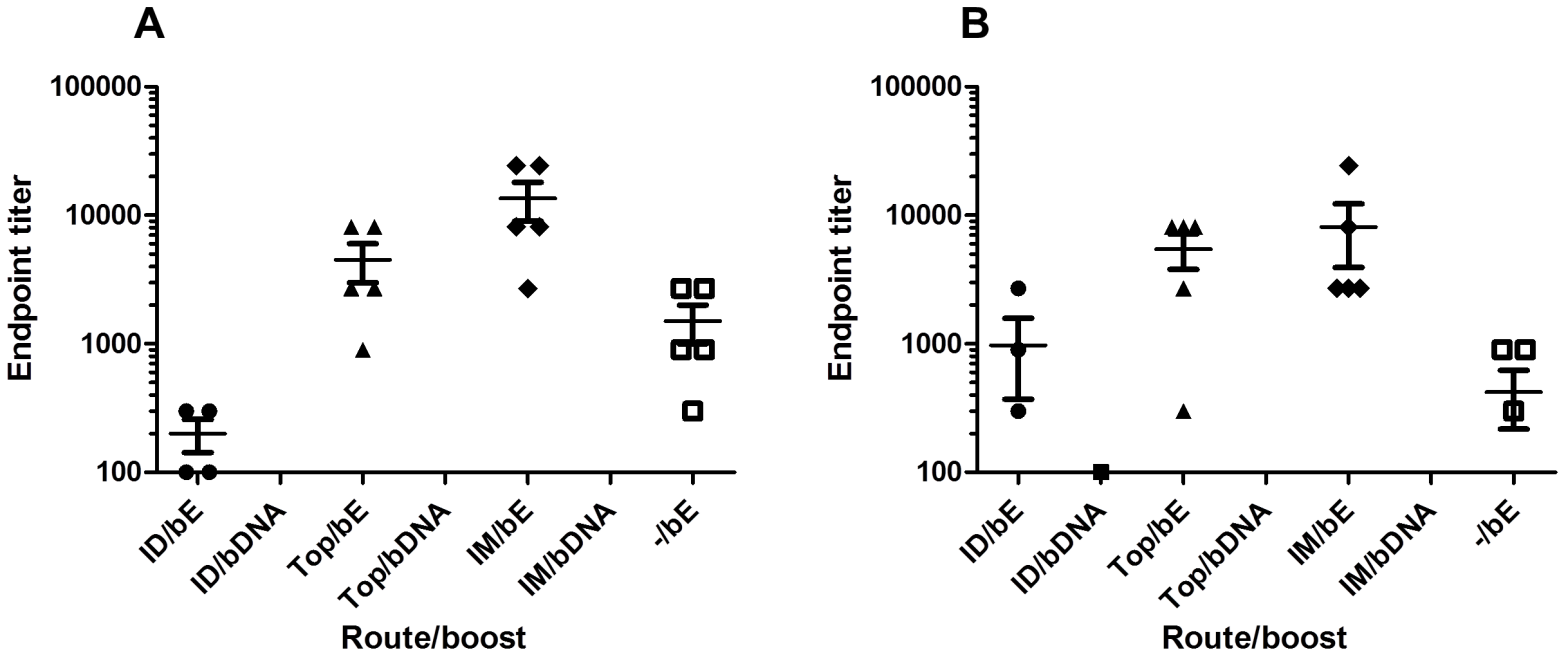
Detection of serum antibody to DIII of the E-protein. IgG1 (A) and IgG2a (B) titers of mice primed ID (4 mice), IM (5 mice), Topically (5 mice) or not primed (−/bE, 5 mice) with WNV-DermaVir nanoparticles and boosted either with WNV-DermaVir nanoparticles (IM) or E-protein combined with Matrix-M1 adjuvant (s.c.). IgG1 (A) and IgG2a (B) titers were determined by ELISA against recombinant DIII. Abbreviations: bE: boost E-protein, bDNA: boost WNV-DermaVir nanoparticles.

In mice, monoclonal antibodies that map to the lateral ridge of domain III (DIII) of the E protein comprise a dominantly neutralizing epitope [40], [44]. As such, we determined the antibody levels against D III after boosting the mice. Intramuscular priming with WNV-DermaVir nanoparticles modestly enhanced the IgG1 titers against DIII as compared to the topical primed mice, although the difference was not statistically significant (Figure 3A). However, the IgG1 titers were resp. 9- and 3-fold higher after IM or topical priming compared to no priming. In comparison, similar IgG2a (Figure 3B) titers against DIII following either IM or topical priming with WNV-DermaVir nanoparticles were observed and they were resp. 19- and 13-fold higher compared to mice that were not primed with WNV-DermaVir nanoparticles. As expected, no antibody response was detected when mice were given a boost with WNV-DermaVir nanoparticles.

**Figure 4:**
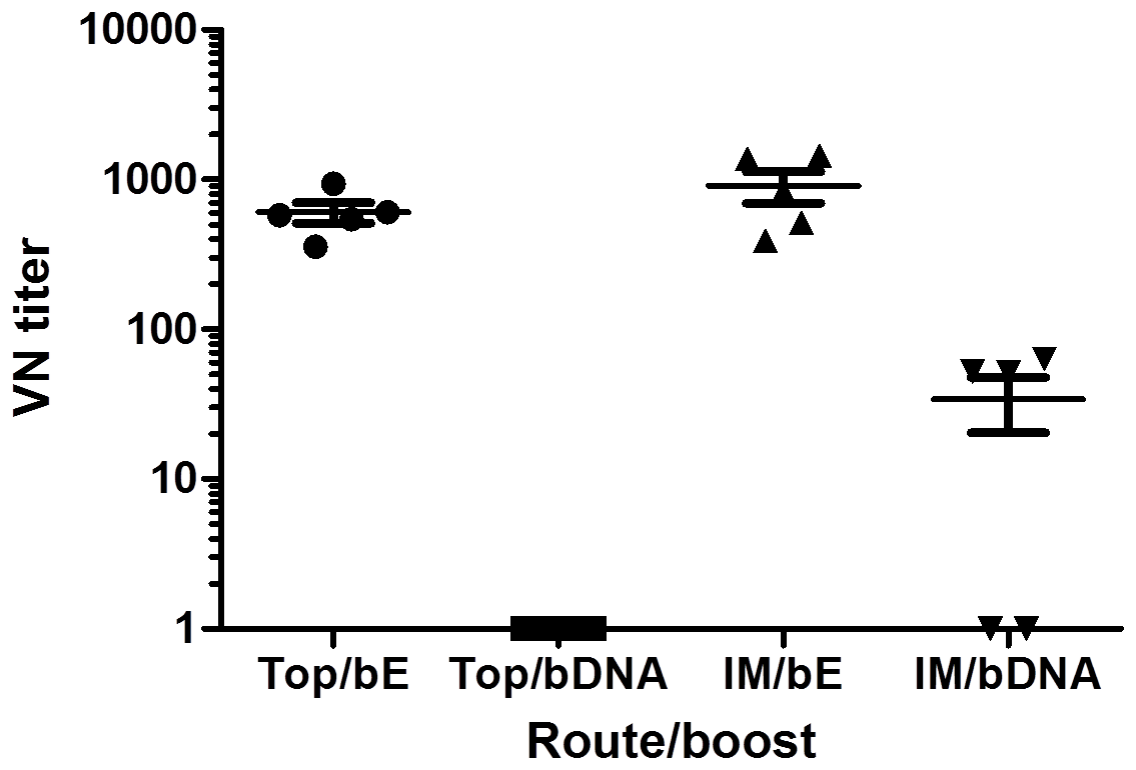
Detection of virus neutralizing antibodies. Five mice were vaccinated IM or topically with WNV-DermaVir nanoparticles and boosted either with WNV-DermaVir nanoparticles (IM) or E-protein combined with Matrix-M1 adjuvant (s.c.). Virus neutralization titers were determined by a focus reduction neutralization assay. Abbreviations: bE: boost E-protein, bDNA: boost WNV-DermaVir nanoparticles.

Next, virus neutralizing titers, which are very important for protection against infection, were determined after vaccination via the best administration routes in mice vaccinated (IM and topical). All the mice primed with WNV-DermaVir nanoparticles and boosted with E-protein had detectable virus neutralizing antibodies while none (topical priming) or three out of five mice (IM priming) raised virus neutralizing antibodies after boosting with WNV-DermaVir nanoparticles (Figure 4).

### Vaccination with WNV-DermaVir Nanoparticles Induces Cellular Immune Responses

**Figure 5:**
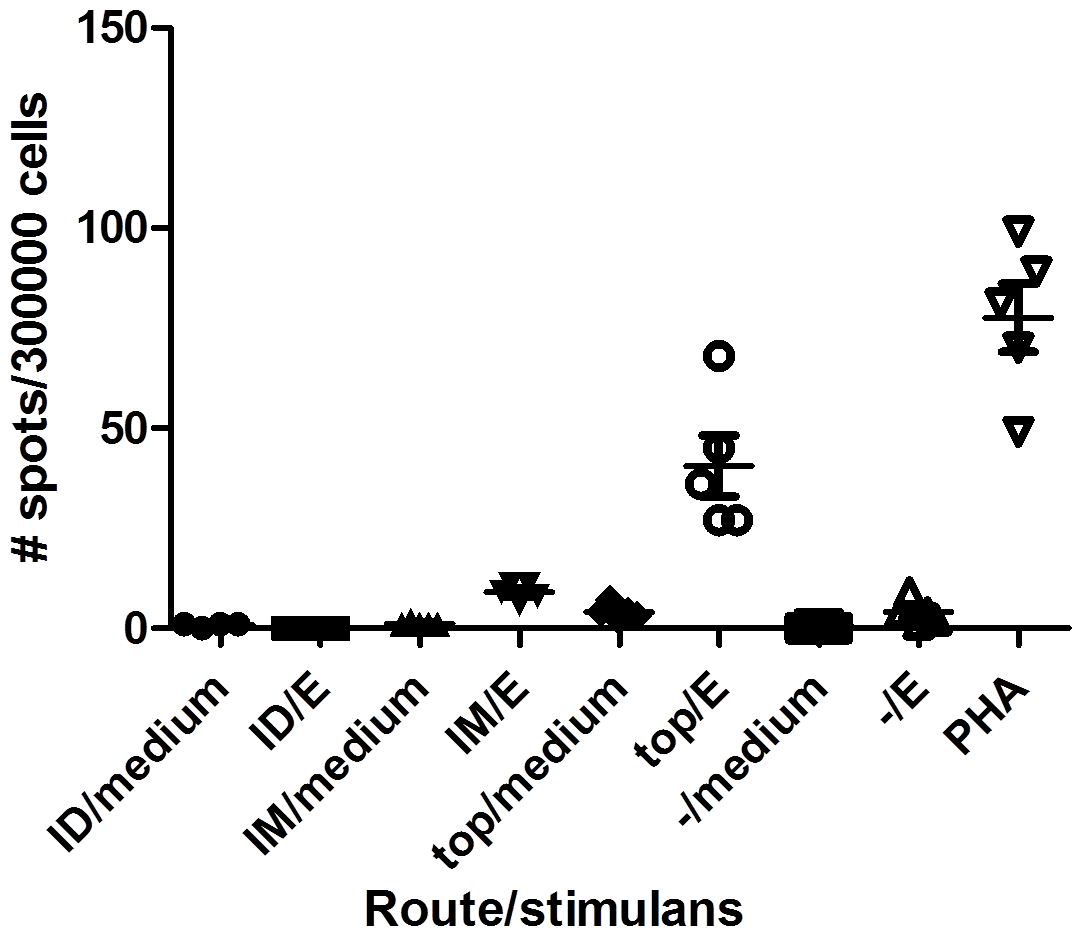
Induction of WNV-E specific IL-4 response by vaccination with WNV-DermaVir nanoparticles followed by a WNV-E protein boost. WNV-E specific IL-4 responses were determined by IL-4 ELISPOT. Splenocytes obtained two weeks after the boost were stimulated with WNV-E protein and the numbers of cells producing IL-4 per 3×10^5^ cells were determined in triplicate. Mice were primed ID (4 mice), IM (5 mice), topically (top, 5 mice) or not (−/, 5 mice) with WNV-DermaVir nanoparticles and boosted s.c. with E-protein combined with Matrix-M1.

To determine whether the vaccination regimes induced a cellular immune response we collected splenocytes two weeks after boosting with WNV-E protein. WNV-E protein specific IL-4 and IFN-γ responses were measured via ELISA and intracellular cytokine staining (ICS), respectively. Mice that were Top or IM primed with WNV-DermaVir nanoparticles had a higher percentage of IL-4 producing splenocytes than mice that received the WNV-DermaVir nanoparticles prime via an ID injection (Figure 5). The difference between the topical group and the ID group was statistically significant (P<0.016, Tukey test). Mice receiving a single injection with E protein (without priming, Figure 4) and mice boosted with WNV-DermaVir nanoparticles (data not shown) did not produce IL-4 positive spots.

**Figure 6:**
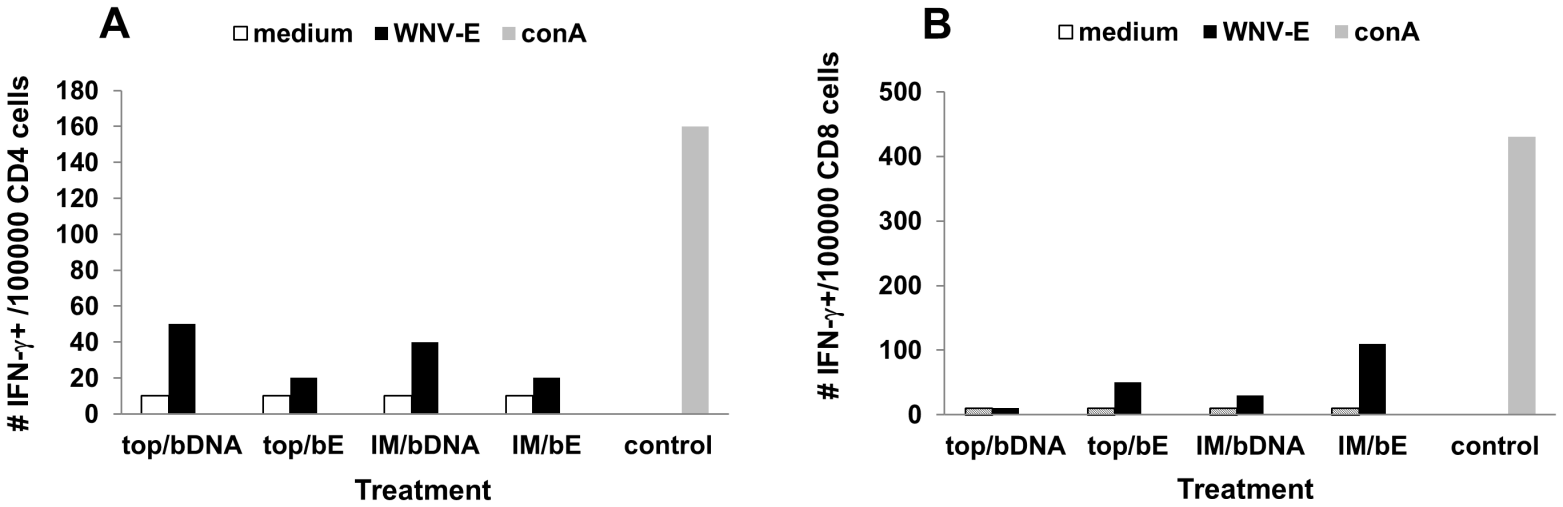
Intracellular IFN-γ production in CD4^+^ (A) and CD8^+^ (B) splenocytes. Five mice were IM or Topically primed with WNV-DermaVir nanoparticles and boosted IM with WNV-DermaVir nanoparticles or s.c. with WNV-E protein combined with Matrix-M1. Splenocytes were pooled per group. Cells were stained for cytokines and surface markers (as indicated in the Methods) and analyzed by flow cytometry. Abbreviations: bE: boost E-protein, bDNA: boost WNV-DermaVir nanoparticles.

Next, the amount of IFN-γ producing CD4^+^ and CD8^+^ splenocytes was determined. The percentage of CD4^+^ cells that produced IFN-γ was 2 to 2,5-fold higher in the mice boosted with WNV-DermaVir nanoparticles compared to the mice boosted with WNV-E protein (Figure 6A). In comparison, in the CD8^+^ population mice boosted with WNV-E protein showed the highest amount of IFN-γ positive cells, and their relative numbers were higher than their respective relative numbers of IFN-γ positive CD4^+^ cells (Figure 6B).

### Heterologous Vaccination Protects against Lethal WNV Infection

**Figure 7:**
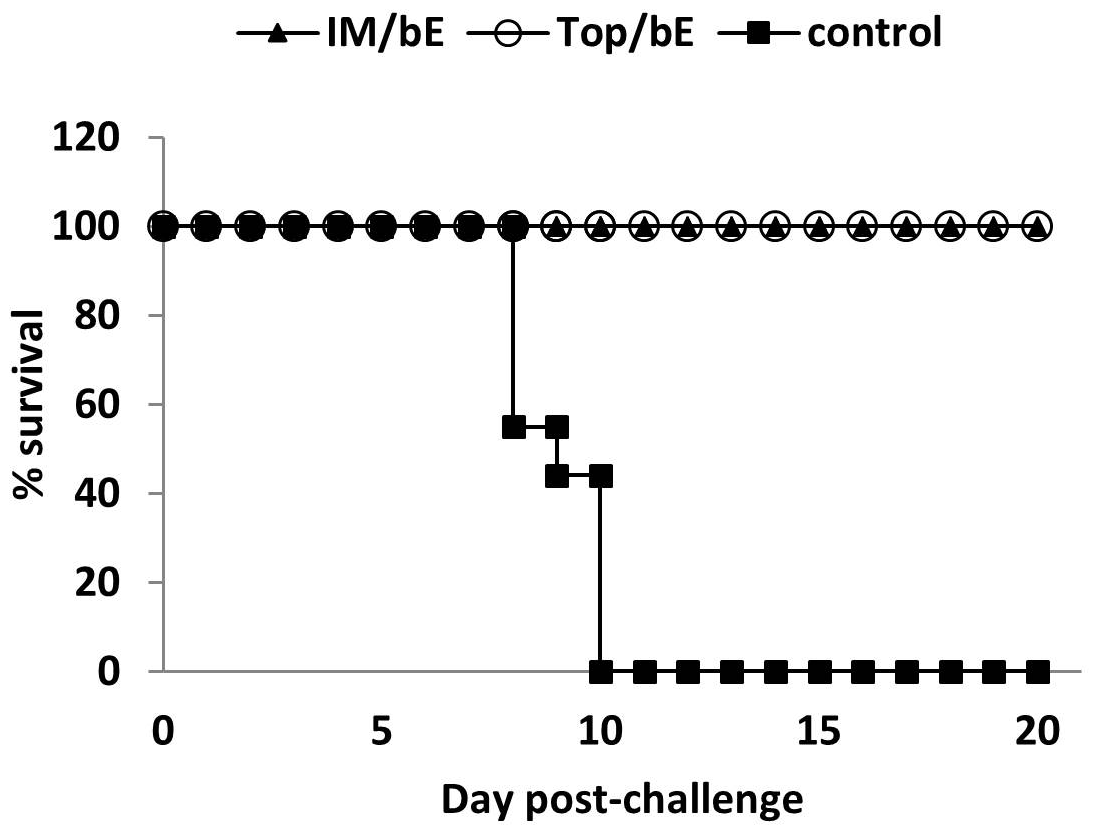
Heterologous prime (WNV-DermaVir nanoparticles) - boost (WNV-E protein) protect mice against a lethal WNV challenge. Two groups of 10 BALB/c mice were vaccinated IM or topically (top) with WNV-DermaVir nanoparticles and boosted with WNV-E protein combined with Matrix-M1 four weeks later. Nine control mice were vaccinated with WNV-DermaVir nanoparticles containing an egfp control plasmid. Two weeks after the boost, the mice were challenged i.p. with a lethal dose of WNV. Abbreviations: bE: boost E-protein.

**Figure 8:**
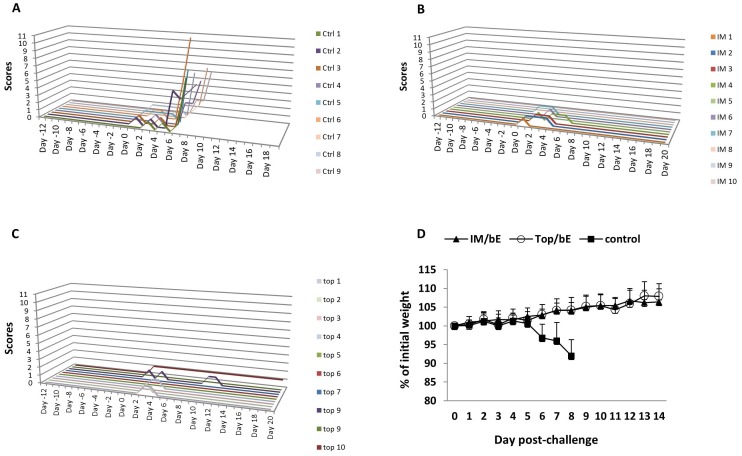
Heterologous prime (WNV-DermaVir nanoparticles) - boost (WNV-E protein) protect mice against morbidity after a lethal WNV challenge. Two groups of 10 BALB/c mice were vaccinated IM or topically (top) with WNV-DermaVir nanoparticles and boosted with WNV-E protein combined with Matrix-M1 four weeks later. In addition, nine control mice were vaccinated with WNV-DermaVir nanoparticles containing an egfp control plasmid. Two weeks after the boost, the mice were challenged i.p. with a lethal dose of WNV. Individual illness scores of mice vaccinated with (A) egfp control plasmid, (B) IM with and (C) topically with WNV-DermaVir nanoparticles. (D) Body weight in percent of individual baseline mean after challenge. Abbreviations: bE: boost E-protein.

Protection against a lethal WNV infection was studied with the two most potent vaccination regimes, i.e. intramuscular or topical priming with WNV-DermaVir nanoparticles followed by subcutaneous boosting with WNV-E protein. All mice primed by either route survived the infection (Figure 7). The survival rates of these groups were significantly higher than that of the control group vaccinated with egfp-DermaVir nanoparticles, in which all of the mice succumbed to the infection (*P*<0.001, Kaplan–Meier). All nine control animals challenged with WNV developed severe disease. There individual total illness scores started to rise by day 7 requiring sacrifice in the following days (Figure 8A). In the WNV-DermaVir vaccinated groups a much lower total score (maximum score of 1) was obtained during the first week after challenge (Figures 8B and 8C). Correspondingly, morbidity as measured by weight loss was observed in the control animals from day 6 until death and was significantly different from the two WNV-DermaVir nanoparticles vaccinated groups on days 7 and 8 (P<0.001, Tukey test) (Figure 8D). However, there was no apparent difference in severity of infection between the two WNV-DermaVir nanoparticles vaccinated groups.

The effect of vaccination on the viral load in blood and brain also was assessed. No viral RNA could be detected in the WNV-DermaVir nanoparticles vaccinated animals whereas varying levels of WNV RNA were detected in all control animals (Table 3).

**Table 3:** Virus load in blood and brain post-infection in vaccinated (10 mice per group) and nine control mice.

Vaccination regime	Blood	Brain
**IM-E**	<80	<80
**Top-E**	<80	<80
**Control**	9322±11081	1.31×10^8^±3.32×10^8^

BALB/c mice were immunized IM or topical with WNV-DermaVir nanoparticles and four weeks later boosted s.c. with WNV-E protein formulated with Matrix-M1. Two weeks after the boost, the mice were challenged i.p. with a lethal dose of WNV. Two days after the challenge virus titers were determined in blood. Viral load in the brain was determined at the day of euthanasia of the moribund animal or at the end of the experiment in the surviving animals (i.e. 20 days post challenge). The number of WNV RNA copies/sample were determined via RT-PCR.

### Safety and Toxicological Evaluation of the WNV-DermaVir Nanoparticles

**Figure 9:**
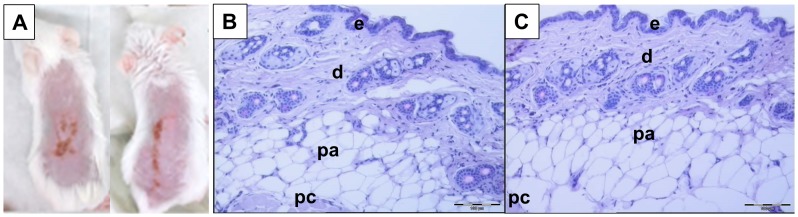
Macro- and microscopical analysis of the skin after topical application of WNV-DermaVir nanoparticles. Panel A shows the macroscopic lesions observed on the back of two representative mice one day after the topical application of the WNV-DermaVir nanoparticles. Panel B and C are H&E- stained skin biopsies of a control animal (B) and an animal that received WNV-DermaVir nanoparticles via topical application. The skin biopsies were taken from the treated area 14 day after topical application of the WNV-DermaVir nanoparticles. The different structures of the skin are noted : epidermis (e), dermis (d), panniculus adiposus (pa) and the panniculus carnosus (pc).

The outermost layer of the epidermis, the stratum corneum, acts as the major skin barrier and limits ingress of foreign agents into the skin. Therefore, before the topical application of the WNV-DermaVir nanoparticles we removed the stratum corneum via tape stripping. Macroscopically, erythema with focal exudation could be noticed shortly after tape stripping and WNV-DermaVir application (figure 9A). No bleeding was observed. After a few days the exudate ceased, the erythema decreased and by two weeks later no macroscopic lesions remained. Additionally, also no long-lasting microscopic lesions or infection were observed two weeks after the topical application of the WNV-DermaVir nanoparticles (Figure 9B and 9C).

A preliminary toxicity study was performed with the WNV-DermaVir nanoparticles using total serum bilirubin and serum alanine transaminase (ALT) levels as markers. Table 4 shows the total serum bilirubin and ALT level 14 days after intramuscular or topical application of the WNV-DermaVir nanoparticles. The concentration of both ALT and total serum bilirubin fell in the normal range for mice [45].

**Table 4:** Toxicity markers measured after IM or topical application of WNV-DermaVir nanoparticles.

	ALT (IU/l)	Total bilirubin (mg/dl)
**IM**	34±5	1.7±0.9
**Top**	41±5	1.5±0.3
**Normal range**	17–77	0.1–1.9

The serum ALT and total serum bilirubin levels were determined 14 days after intramuscular or topical administration of the WNV-DermaVir nanoparticles in five mice per group.

## Discussion

Vaccination remains the single most effective method for the prevention of infectious diseases. Despite this, there still exists no licensed human WNV vaccine although effective vaccines are available commercially for horses and exotic birds. In this study, we condensed a plasmid DNA encoding a secreted form of the ectodomain of the WNV-E protein with linear polyethyleneimine that contained covalently bound mannose residues (lPEIm). We called the resulting nanoparticles WNV-DermaVir nanoparticles and they had an average size of 114 nm and a slightly positive zeta potential of about 8 mV. The low zeta potential of these nanoparticles is most likely due their low N/P ratio, i.e. 4. These WNV-DermaVir nanoparticles combine the strong compaction capacity and intrinsic endosomolytic activity of lPEI [32] with an APC targeting ligand, mannose [36]. WNV-DermaVir nanoparticles were administered by three different routes (ID, IM or topical) to mice, and the humoral and cellular immune response was evaluated. WNV-DermaVir nanoparticles alone did not induce a humoral immune response by themselves. A similar observation was found by Lisziewicz *et al*, who failed to detect antibody responses in macaques despite five repeated immunizations with DermaVir nanoparticles encoding HIV-antigens [29]. However, using a heterologous DNA prime/protein boost immunization strategy, we showed that antibody titers against the E-protein can be induced using these WNV-DermaVir nanoparticles. The antibody levels obtained via this strategy were higher compared to those in mice receiving a single injection of E-protein. This demonstrates that memory B cells were pre-sensitized by the WNV-DermaVir nanoparticles, although apparently few short-lived or long-lived antibody-secreting plasma cells were generated after priming. It is possible that the antigen expression in the local somatic cells (e.g. myocytes, keratinocytes), which serve as antigen reservoirs for conventional DNA vaccines, was too low to induce a strong high-affinity antibody response but was sufficient to establish non-antibody secreting memory B cells, which then could be readily activated after homologous antigen boost with a potent adjuvant. The difference in immune response between the ID and topical administration, two delivery routes that target the skin as immunization site, might be due to the fact that topical application resulted in greater skin injury and local inflammation as seen in figure 7. This has been shown to stimulate the recruitment of Langerhans cells from skin to draining lymph nodes [46], [47] and result in the production of cytokines [48]. In this way, the physical stress associated with the delivery method acts as a type of immunological adjuvant. In addition, protein expression after ID administration may be lower than after injection into the muscle, which might explain the difference in antibody titers between both administration routes. Since the most potent inhibitory antibodies recognize neutralizing epitopes on DIII [49], [50], we also examined whether DIII-specific antibodies accumulated in serum from protein boosted mice that received the DNA prime topical or IM. Indeed, we detected antibodies directed against DIII of the E-protein regardless of the route of priming but only after a protein boost.

The ratio of IgG1/IgG2a for each administration route indicated that a Th2-like response was induced. Intradermal injection and topical applications have been shown to elicit a humoral response characterized by a Th2-type response [51] whereas injection into the muscle results more in a Th1 response [52], [53]. We also found the lowest IgG1/IgG2a ratio when the WNV-DermaVir nanoparticles prime was given IM. This result was confirmed by IL-4 and IFN-γ cytokine assays. A moderate (topical priming) to weak (IM priming) IL-4 response was observed after DNA prime/protein boost vaccination. In addition, only a very weak CD4^+^ IFN- γ production was observed in the same groups. On the contrary, the mice that received twice WNV-DermaVir nanoparticles developed no IL-4 response, but demonstrated a higher IFN- γ production by CD4^+^ cells than mice that were vaccinated via the DNA prime/protein boost approach. Smitha *et al.*, who vaccinated mice via topical application of lPEIm nanoparticles containing a plasmid DNA encoding a Fasciola gigantica fatty acid binding protein, also found significant expression of IFN-γ but insignificant levels of IL-4 [54].

Next, we analyzed the IFN-γ expression by CD8^+^ T cells. We mainly observed a IFN-γ response in the groups boosted with adjuvanted E-protein. Since the plasmid in the vaccine encodes for secreted ectodomain of the E-protein it was not unexpected to find a relatively weak CD8^+^ response. Indeed, by comparing three different OVA plasmids encoding a secreted form, a cytoplasmatic form and a membrane-bound form, Boyle *et al* found that the secreted form was more potent than other forms in imitating humoral immune responses, whereas the cytoplasmatic form was most potent to induce CTL responses [55]. The CD8^+^ response that we detected after protein boost might be attributed largely to the adjuvant Matrix-M1. This adjuvant is related to another saponin-based adjuvant named ISCOMATRIX, which has been shown to promote CD8^+^ T cell cross-priming. Indeed, MHC class I cross-presentation by CD8α^+^ DCs was enhanced up to 100-fold when antigen was formulated with ISCOMATRIX adjuvant [56]. Cross-presentation enable DCs to activate CD8^+^ T cells by uptake, processing and MHC class-I restricted epitope presentation of exogenous antigen e.g. soluble proteins, immune complexes and pathogen-associated antigens.

The importance of an antibody response against WNV to control WNV infections has been described [57]. IgM associated antibodies limit viral dissemination in the host early after onset of clinical signs [58]. IgG appear a few days later and they effectively clear the virus and mediate long-term protective immunity [59]. In addition, the T cell response also plays a crucial role in recovery, particular in virus clearance [60]. As our heterologous prime-boost generated both humoral and cellular immune response, we evaluated its efficacy in a challenge model in mice. The heterologous vaccination regime resulted in a complete virus clearance in the blood and brain of immunized animals. Mice were fully protected against a lethal infection and in addition never demonstrated body weight loss during the 20 days observation period after challenge.

To ensure the safe administration of vaccines to humans, vaccines are evaluated in preclinical safety assessment studies that aim at identifying the potential toxicities associated with their administration. Therefore we determined in mice the total serum bilirubin and serum Alanine Transaminase (ALT) levels. The determination of serum bilirubin is an important marker for the diagnosis of several diseases; elevated levels of bilirubin are strongly associated with hemolysis, blockage of the biliary tract, and liver disease [61]. Elevation of ALT levels is an indication of liver damage and has been associated with liver injury [61]. Importantly, we did not observe elevations in serum levels of both toxicity indicators.

In conclusion, our data suggests that DNA priming with WNV-DermaVir nanoparticles and boosting with adjuvanted WNV-E protein enhanced protective antibody and CD8+ T cell responses and therefore appears an excellent safe candidate for further studies in other animals.
