# Correction: Vaccination of Mice Using the West Nile Virus E-Protein in a DNA Prime-Protein Boost Strategy Stimulates Cell-Mediated Immunity and Protects Mice against a Lethal Challenge

**DOI:** 10.1371/journal.pone.0091631

**Published:** 2014-02-28

**Authors:** The PLOS ONE Staff

The last sentence of the “WNV DNA Vaccine, Control Plasmid and E-protein” section of the Materials and Methods should read: “For protein booster vaccine formulation the E-protein was adjuvanted with 10 μg Matrix-M1 adjuvant (Novavax AB, Uppsala, Sweden).”

In addition, the following sentence should be inserted as the last sentence of the “Vaccination Procedures” section of the Materials and Methods: “Mice were boosted four weeks later either via an IM injection of the same does of WNV-DermaVir nanoparticles or via subcutaneous (s.c.) injection of recombinant E-protein ([28]) formulated with Matrix-M1 (a kind gift from Novavax AB, Uppsala, Sweden).”
